# New Measure of Insulin Sensitivity Predicts Cardiovascular Disease Better than HOMA Estimated Insulin Resistance

**DOI:** 10.1371/journal.pone.0074410

**Published:** 2013-09-30

**Authors:** Kavita Venkataraman, Chin Meng Khoo, Melvin K. S. Leow, Eric Y. H. Khoo, Anburaj V. Isaac, Vitali Zagorodnov, Suresh A. Sadananthan, Sendhil S. Velan, Yap Seng Chong, Peter Gluckman, Jeannette Lee, Agus Salim, E. Shyong Tai, Yung Seng Lee

**Affiliations:** 1 Saw Swee Hock School of Public Health, Yong Loo Lin School of Medicine, National University of Singapore and NUHS, Singapore, Singapore; 2 Department of Obstetrics and Gynaecology, Yong Loo Lin School of Medicine, National University of Singapore and NUHS, Singapore, Singapore; 3 Department of Medicine, Yong Loo Lin School of Medicine, National University of Singapore and NUHS, Singapore, Singapore; 4 Department of Endocrinology, Tan Tock Seng Hospital, Singapore, Singapore; 5 Singapore Institute for Clinical Sciences, Agency for Science, Technology and Research, Singapore, Singapore; 6 Neuroscience and Behavioral Disorders Program, Duke-NUS Graduate Medical School, Singapore, Singapore; 7 School of Computer Engineering, Nanyang Technological University, Singapore, Singapore; 8 Singapore BioImaging Consortium, NUS-A*STAR, Singapore, Singapore; 9 Clinical Imaging Research Centre, NUS-A*STAR, Singapore, Singapore; 10 Department of Paediatrics, Yong Loo Lin School of Medicine, National University of Singapore and NUHS, Singapore, Singapore; Monash University, Australia

## Abstract

**Context:**

Accurate assessment of insulin sensitivity may better identify individuals at increased risk of cardio-metabolic diseases.

**Objectives:**

To examine whether a combination of anthropometric, biochemical and imaging measures can better estimate insulin sensitivity index (ISI) and provide improved prediction of cardio-metabolic risk, in comparison to HOMA-IR.

**Design and participants:**

Healthy male volunteers (96 Chinese, 80 Malay, 77 Indian), 21 to 40 years, body mass index 18−30 kg/m^2^. Predicted ISI (ISI-cal) was generated using 45 randomly selected Chinese through stepwise multiple linear regression, and validated in the rest using non-parametric correlation (Kendall's tau τ). In an independent longitudinal cohort, ISI-cal and HOMA-IR were compared for prediction of diabetes and cardiovascular disease (CVD), using ROC curves.

**Setting:**

The study was conducted in a university academic medical centre.

**Outcome measures:**

ISI measured by hyperinsulinemic euglycemic glucose clamp, along with anthropometric measurements, biochemical assessment and imaging; incident diabetes and CVD.

**Results:**

A combination of fasting insulin, serum triglycerides and waist-to-hip ratio (WHR) provided the best estimate of clamp-derived ISI (adjusted R^2^ 0.58 versus 0.32 HOMA-IR). In an independent cohort, ROC areas under the curve were 0.77±0.02 ISI-cal versus 0.76±0.02 HOMA-IR (*p*>0.05) for incident diabetes, and 0.74±0.03 ISI-cal versus 0.61±0.03 HOMA-IR (*p*<0.001) for incident CVD. ISI-cal also had greater sensitivity than defined metabolic syndrome in predicting CVD, with a four-fold increase in the risk of CVD independent of metabolic syndrome.

**Conclusions:**

Triglycerides and WHR, combined with fasting insulin levels, provide a better estimate of current insulin resistance state and improved identification of individuals with future risk of CVD, compared to HOMA-IR. This may be useful for estimating insulin sensitivity and cardio-metabolic risk in clinical and epidemiological settings.

## Introduction

Insulin resistance, or reduced insulin sensitivity, is the key pathophysiologic defect in type 2 diabetes mellitus, metabolic syndrome and cardiovascular disease [1], [2], [3], [4]. Accurate assessment of insulin sensitivity helps to identify individuals at increased risk of these diseases, and may help target preventive and therapeutic efforts more effectively. The “gold standard” method for the assessment of insulin sensitivity is the hyperinsulinemic euglycemic clamp. This method estimates insulin sensitivity directly by determining peripheral glucose disposal rate during the steady-state of hyperinsulinemia when blood glucose is maintained at euglycemic levels by an exogenous glucose infusion [5]. Although it is widely-accepted as the reference method, the euglycemic clamp is costly, labor- and time-consuming. Thus, this approach is usually confined to research settings and is not feasible in population studies.

The most commonly used surrogate measure of insulin resistance is the homeostatic model of insulin resistance (HOMA-IR) [6], which is a computation based on fasting insulin and glucose values. It has moderate correlations (r = −0.38 to −0.66) with insulin sensitivity measured by the clamp technique [7], [8]. Since insulin sensitivity is influenced by and associated with other factors such as excess adiposity and dyslipidemia [4], [9], [10], [11], [12], [13], [14], [15], we hypothesize that a combination of simple anthropometric and biochemical parameters might provide a better estimate of insulin sensitivity than HOMA-IR. In this study, we derive an estimate of insulin sensitivity based on anthropometric and routine biochemical parameters. We examine if this derived measure of insulin sensitivity offers any advantage over HOMA-IR in identifying individuals with insulin resistance and risk of future cardio-metabolic events.

## Subjects and Methods

### Ethics statement

For the Singapore Adult Metabolism Study (SAMS), ethical approval was obtained from the National Healthcare Group Domain Specific Review Board prior to conduct of the study, and written informed consent was obtained from all participants.

For the independent longitudinal cohort, ethics approval was obtained from two Institutional Review Boards (National University of Singapore and Singapore General Hospital). Written informed consent was obtained before conduct of the study.

### Singapore Adult Metabolism Study

Healthy male volunteers, aged 21–41 years and with body mass index (BMI) between 18.5–30 kg/m^2^, were invited to participate in SAMS. Individuals with known hypertension, diabetes mellitus, dyslipidemia, ischemic heart disease, epilepsy or any medication that might affect insulin sensitivity (eg, corticosteroids) were excluded. Also excluded were individuals with recent changes in or attempts to change body weight, bleeding diathesis, inaccessible veins, recent investigational medicine use, or contraindications to magnetic resonance imaging (MRI).

Anthropometric measures included height, weight, waist and hip circumference, measured twice and the average taken, as well as skinfold thicknesses at four sites (biceps, triceps, subscapular and suprailiac), measured in triplicate. The waist-to-hip ratio (WHR) was calculated by dividing waist circumference (in cm) by hip circumference (HC, in cm). Percentage body fat was derived from the skinfolds measured using the following formula [16]: Body fat % = 36.7 * log (sum of 4 skinfolds: triceps, biceps, subscapular and suprailiac) −39.5.

All subjects also underwent a whole body Dual Energy X-ray Absorptiometry (DEXA) scan (a single Hologic Discovery Wi densitometer, Hologic, Inc, Massachusetts, USA) to estimate total fat mass, and magnetic resonance imaging (MRI) of the abdomen (3T Trio, Siemens AG, Medical Solutions, Germany) from lumbar segments T12 to L5, to quantify subcutaneous and visceral fat.

#### Hyperinsulinemic euglycemic glucose clamp

Insulin sensitivity was assessed using the euglycemic, hyperinsulinemic clamp technique as previously described [17]. Subjects were instructed to fast overnight (10–12 hours). On the following morning, two cannulae were inserted, one for infusion of 20% dextrose solution and insulin, and the second into a contralateral vein for blood sampling. After baseline blood samples were taken, insulin was infused at a fixed-rate of 40 mU/m^2^ body surface area/minute for the duration of 120 minutes. Blood glucose concentrations were measured at 5-minute intervals and the infusion rate of 20% dextrose solution was adjusted to maintain blood glucose concentration at 5.0 mmol/L (90 mg/dL) throughout the clamp period. Blood glucose was measured using an enzyme biosensor glucose analyzer (YSI 2300 STATPLUS, YSI Incorporated, Life Sciences, USA). The insulin sensitivity index (ISI-clamp) was calculated using the mean glucose disposal rate during the final 30 minutes of the clamp experiment (mg/min/mU/kg lean body mass).

#### Biochemical analyses

Blood samples were collected from all participants in the morning after a 10-hour overnight fast. Venous blood was drawn and collected in plain and fluoride oxalate tubes and stored at 4°C for a maximum of 4 hours prior to processing. All biochemical analyses were carried out at the National University Hospital Referral Laboratory, which is accredited by the College of American Pathologists. Serum total cholesterol, triglyceride (TG), and high density lipoprotein cholesterol levels were measured using an automated auto-analyzer (ADVIA 2400, Siemens Medical Solutions Diagnostics, USA). Low density lipoprotein cholesterol levels were calculated using the Friedewald formula [18]. Insulin level was analyzed using a sandwich assay with 2 monoclonal mouse anti-insulin antibodies (ADVIA Centaur, Siemens Medical Solutions Diagnostics, USA).

### Independent longitudinal cohort

We looked at data from participants from two cross-sectional surveys, the National Health Survey (NHS 1992) [19] and the National Health Survey (NHS 1998) [20], who repeated the health survey between 2004 and 2007. Briefly, both studies were a random sample of from the Singapore population, with disproportionate sampling stratified by ethnicity to increase the number of the minority ethnic groups (Malays and Asian Indians). Details of participant characteristics and biochemical analysis have been published previously [19], [20].

A total of 6,302 subjects who participated in one of the NHS (1992 or 1998), were recontacted between 2004 and 2007 for follow up examination. Of these 4,023 subjects had complete data for anthropometric parameters, biochemical measures and diabetes mellitus (DM) and cardiovascular disease (CVD) status at baseline (NHS 1992 or 1998) and follow-up. Subjects with DM (n = 202) or CVD (n = 71) at baseline were excluded. Thus, a total of 3,750 subjects were included for the final analysis.

### Definitions

Diabetes mellitus was defined using fasting plasma glucose based on ADA criteria, as well as history of diabetes/diabetes medication. Cardiovascular disease in NHS 1992 was defined as history of ischaemic heart disease/angina/stroke. Cardiovascular disease in NHS 1998 was defined as history of angina/stroke.

Cardiovascular disease at follow up was defined as history of ischaemic heart disease/blockage of coronary arteries/angina/stroke. Homeostatic Model of Assessment of Insulin Resistance (HOMA-IR) was calculated as [I_0_ (µU/mL) × G_0_ (mmol/L)] ÷22.5 [6], I_0_ =  fasting insulin, G_0_ = fasting glucose.

### Statistical analysis

A total of 253 subjects (96 Chinese, 80 Malay, and 77 Indian) from SAMS with complete clamp, anthropometric and biochemical data were included in the analysis. All variables were checked for normality and log-transformed to improve normality assumptions as necessary. A sub-sample was drawn from the Chinese subjects in the study using the random sampling option in the statistical software to select approximately half of the subjects available. In this sub-sample of individuals, Pearson's correlation coefficients were used to identify significant associations between anthropometric, biochemical and imaging measures with ISI-clamp. Stepwise multiple linear regression was performed to identify predictors of ISI-clamp, and to generate the prediction equation (ISI-cal). Alternative models were evaluated by comparing the total variation explained by the models. Findings were confirmed using robust regression. Bootstrapping was used to generate bias corrected and accelerated confidence intervals for the beta coefficients.

The equation was validated by comparing non-parametric correlation coefficients (Kendall's tau-a) between various estimates of insulin sensitivity and ISI-clamp, and testing for significant differences between the correlation coefficients (using the *lincom* command in STATA). Lin's concordance coefficient was also calculated to estimate agreement between ISI-clamp and ISI-cal. Receiver operating characteristic (ROC) curves were used to compared the predictive function of different methods in identifying individuals with insulin resistance, as defined by the lowest tertile of insulin sensitivity by ISI-clamp, i.e. ISI ≤6.94 mg min^−1^ kg lean mass^−1^ mU insulin^−1^.

We then examined the ability of the derivative ISI-cal in predicting cardio-metabolic events in an independent longitudinal cohort. ISI-cal and HOMA-IR were calculated for the baseline cohorts (NHS 1992 and NHS 1998). ROC curves were used to compare predictive function between HOMA-IR and ISI-cal in identifying new onset of DM and CVD in the follow – up cohort. Youden's index was used to identify cut-off values with optimum specificity and sensitivity. McNemar's statistic was used to test for differences in sensitivity and specificity between ISI-cal and metabolic syndrome definition in predicting CVD.

The random sample was generated using the random sampling facility in SPSS (Version 19, IBM Statistics, USA). All other statistical analyses were performed using Stata 10.0 for Windows (Stata Corporation, College Station, Texas, USA). Data are presented as means ± SD unless stated otherwise. All statistical tests were two-sided, with any *p<*0.05 being considered significant.

## Results

Table 1 summarizes the characteristics of subjects from SAMS and the independent cohort.

**Table 1:** Baseline characteristics of subjects in SAMS and an independent cohort.

	SAMS study				Independent cohort				
	Prediction group (N = 45)		Validation group (N = 208)		At baseline (N = 3750)		At follow up		
Variable	Mean	SD	Mean	SD	Mean	SD	N	Mean	SD
Age	28	6.56	27	5.16	38	11.1	3748	47	11.11
Ethnicity, N, %							3661		
Chinese	45	100	51	24.52	2,616	69.76		2,558	69.87
Malay	0	0	80	38.47	608	16.21		594	16.23
Indian	0	0	77	37.02	526	14.03		509	13.9
Gender, N %							3661		
Male	45	100	208	100	1,748	46.6		1,706	46.6
Female	0	0	0	0	2,002	53.4		1,955	53.4
BMI	23.4	2.96	24.4	3.30	23.1	3.95	3745	23.7	4.31
FPG	4.76	0.38	4.64	0.48	5.41	0.5	3750	4.93	1.16
Total cholesterol	4.94	0.79	4.92	0.98	5.39	1.05	3745	5.22	0.93
Triglycerides	1.19	0.69	1.14	0.65	1.38	1.27	3745	1.33	0.84
Fasting insulin	9.47	4.93	11.67	7.75	7.53	5.48	3655	7.77	6.48
WHR	0.85	0.05	0.85	0.05	0.81	0.08	3655	0.85	0.08
ISI-clamp	10.38	4.12	9.8	5.1					

BMI – body mass index, FPG – fasting plasma glucose, WHR – waist hip ratio.

### Generation of prediction equation

In the random sample of 45 Chinese subjects, various anthropometric and biochemical measures were associated with ISI-clamp on univariate analysis: BMI (r = −0.45, *p* = 0.002), waist circumference (r = −0.59, *p*<0.001), WHR (r = −0.66, *p*<0.001), skinfold-derived body fat% (r = −0.63, *p*<0.001), DEXA derived total body fat% (r = −0.55, *p*<0.001), serum triglycerides (r = −0.46, *p* = 0.001), fasting insulin (r = −0.59, *p*<0.001), MRI measured visceral (VAT, r = −0.58, *p*<0.001) and subcutaneous (SAT, r = −0.56, *p*<0.001) adipose tissue.

To identify which set of variable(s) best explained the variation in ISI-clamp, we examined several combinations of these variables in separate regression models. A model with WHR (ΔR^2^  = 0.42), I_0_ (ΔR^2^  = 0.13) and TG (ΔR^2^  = 0.04) best predicted insulin sensitivity (total adjusted R^2^  = 0.58), over models with I_0_ alone (adjusted R^2^  = 0.33), and HOMA-IR alone (adjusted R^2^  = 0.32). Replacing WHR with various MRI-derived fat measures, VAT (adjusted R^2^  = 0.52), SAT (adjusted R^2^  = 0.51), VAT/SAT ratio (adjusted R^2^  = 0.46), VAT over hip circumference (adjusted R^2^  = 0.55), SAT over HC (adjusted R^2^  = 0.52), did not improve the adjusted R^2^ over the model based on WHR, I_0_ and TG. Neither did replacing fasting insulin with other surrogate measures of insulin resistance, HOMA-IR (adjusted R^2^  = 0.55) or QUICKI (adjusted R^2^  = 0.56). There was no multi-collinearity in the final model (VIF <1.5, tolerance 0.75). The relationship between these variables and ISI-clamp was confirmed using robust regression, and confidence intervals for the coefficients obtained by boot-strapping (Table S1). The prediction equation finally derived was given as ISI-cal  =  exp(2.65 – (2.63*ln [WHR]) – (0.39*ln [I_0_])- (0.21*ln [TG])) and simplified to ISI-cal  = 14.15* (WHR)^−2.63^ * (I_0_)^−0.39^ * (TG)^−0.21^.

### Validation within SAMS

The prediction equation, ISI-cal, was validated using the remaining 208 subjects from SAMS. Overall, the correlation between ISI-clamp and ISI-cal (Kendall's tau τ = 0.42, *p*<0.001) was stronger than the correlation between ISI-clamp and HOMA-IR (τ = −0.37, *p*<0.001) (*p*_(comparison)_  = 0.045). The correlation coefficients for ISI-cal and HOMA-IR, for Chinese (0.43 versus  = −0.39) and Indians (0.34 versus  = −0.32) were not significant, except for Malays (0.47 versus  = −0.38, *p*_(comparison)_  = 0.03). Lin's concordance coefficient between ISI-clamp and ISI-cal was 0.53 for the whole group, 0.46 for Chinese, 0.6 for Malay and 0.42 for Indian.

**Figure 1:**
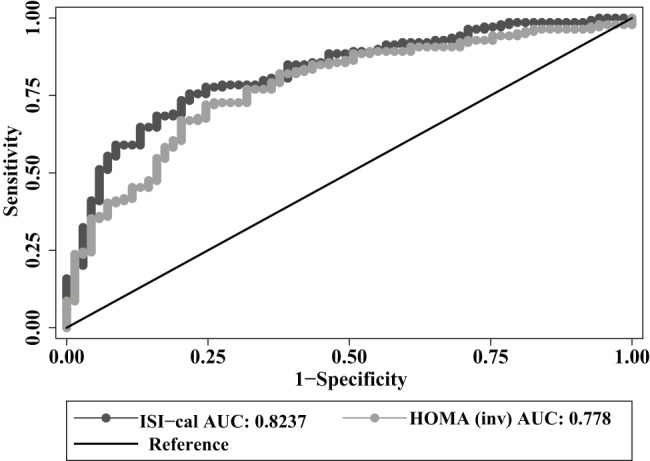
Comparison of ISI-cal with HOMA-IR in validation group from the SAMS study. N = 208; P = 0.024 HOMA-IR – homeostatic model of insulin resistance, ISI-cal – calculated insulin sensitivity, SAMS – Singapore Adult Metabolism study.

The predictive function of these indices in identifying individuals with low insulin sensitivity was tested using ROC curves, with the ISI cutoff set at 6.94 mg min = ^−1^ kg lean mass^−1^ mU insulin^−1^ (the lowest tertile of insulin sensitivity in the group). For this comparison, we used inverse values of HOMA-IR to indicate insulin sensitivity. ISI-cal had a significantly larger area under the ROC curve compared to HOMA-IR (0.82 versus 0.78, *p* = 0.024) (Figure 1).

### Prediction of cardiometabolic events in an independent longitudinal cohort

In the longitudinal cohort (with 6 to 15 years of follow-up), there were 99 individuals who subsequently developed CVD (70 with ischaemic heart disease, 29 with stroke or transient ischaemic attack, and 7 with both), and 213 who developed DM, among those without DM or CVD at baseline. We compared the ability of ISI-cal (inverse) and HOMA-IR in identifying these individuals using ROCs. ISI-cal and HOMA-IR had similar AUCs in predicting DM (Table 2). However, ISI-cal had a significantly larger AUC compared to HOMA-IR in predicting CVD. These findings were replicated across all three ethnic groups and in both genders (Table 2). ISI-cal showed better discrimination for both ischaemic heart disease (ROC ISI-cal 0.75±0.03 vs HOMA-IR 0.63±0.04, *p*<0.001) and stroke (ROC ISI-cal 0.73±0.04 vs HOMA-IR 0.58±0.05, *p*<0.001).

**Table 2:** Prediction of cardio-metabolic events at follow up using ISI-cal and HOMA-IR in the independent cohort.

New Event at follow up (N^a^ = 3750)	HOMA-IR ROC area	ISI-cal (inversed) ROC area	P
Diabetes (n^b^ = 213)	0.76 (0.02)	0.77 (0.02)	NS
CVD, including stroke (n^b^ = 99)	0.61 (0.03)	0.74 (0.03)	<0.001
Chinese (n^b^ = 55)	0.64 (0.04)	0.75 (0.04)	<0.001
Malay (n^b^ = 14)	0.57 (0.08)	0.69 (0.07)	0.03
Indian (n^b^ = 30)	0.49 (0.06)	0.68 (0.05)	<0.001
Male (n^b^ = 78)	0.62 (0.03)	0.7 (0.03)	<0.001
Female (n^b^ = 21)	0.54 (0.08)	0.65 (0.07)	0.02

CVD – cardiovascular disease, HOMA-IR – homeostatic model of insulin resistance, ISI-cal – calculated insulin sensitivity.

a – N– total number of subjects with follow up; b – number of positive events at follow up.

Using Youden's index, the optimal ISI-cal cut-off for predicting DM was 9.3 with sensitivity and specificity of 71%, and for predicting CVD was 9.23 with a sensitivity of 71% and specificity of 70%. The optimal cut-offs for HOMA-IR in this population was 1.99 for DM with sensitivity 70% and specificity 71%, and 1.59 for CVD with sensitivity 64% and specificity 54%. These results did not change materially when we repeated the analysis by cohort of origin (NHS 92, NHS 98) (data not shown).

We also examined whether ISI-cal and metabolic syndrome, as defined by the NCEP ATP III criteria, performed comparably in predicting CVD using McNemar's statistic. We did this to verify whether the gain in predictive accuracy over HOMA-IR was due to improved estimation of the insulin resistance state, or due to the inclusion of triglycerides and WHR, which are independent risk factors for CVD. ISI-cal had a significantly higher sensitivity (70%) compared to metabolic syndrome (25%; *p*<0.001), but also lower specificity (71% ISI-cal vs 92% metabolic syndrome, *p*<0.001). We also ran a logistic regression to evaluate if ISI-cal was associated with future CVD events independent of metabolic syndrome, and found that the association between CVD and ISI-cal was highly significant (OR 4.7, 95% CI 2.9–7.4, *p*<0.001), even when metabolic syndrome was in the model (OR 1.9, 95% CI 1.1–3.1, *p* = 0.02). There was no collinearity between ISI-cal and metabolic syndrome in the model (VIF 1.2, tolerance 0.8).

## Discussion

We derived a prediction equation of insulin sensitivity based on three easily obtainable parameters, namely, waist-to-hip ratio, fasting triglycerides and insulin. This prediction equation correlated well with clamp-measured insulin sensitivity in a validation group that was not used to generate the estimate. In a prospective cohort, the prediction equation performed as well as HOMA-IR in identifying individuals at risk of DM, but significantly better than HOMA-IR in identifying individuals at risk of CVD. This was true for all ethnic groups and for both genders.

Fasting insulin, fasting triglyceride and central adiposity are known to be associated with insulin sensitivity; however a combination of these parameters has not been used to estimate insulin sensitivity. McLaughlin et al have shown that TG or TG-high density lipoprotein ratio was as strongly associated with insulin resistance as fasting insulin levels. They were also the lipid parameters that best correlated with insulin resistance [13], [15]. The equation that we have proposed is similar to the equation described by McAuley et al except that we have included a measure of central obesity, which has greater association with development of insulin resistance and CVD compared to BMI [21], [22], [23]. McAuley et al proposed two prediction equations for insulin sensitivity, one using TG and fasting insulin, and another with inclusion of BMI, using a sample of 178 normoglycemic subjects. They observed that when TG was included but not BMI, there was a a modest but significant increase in sensitivity compared to insulin alone in predicting insulin sensitivity [24]. In our study, addition of WHR instead of BMI increased the ability of our prediction equation to estimate insulin sensitivity. We have further demonstrated the efficacy of this prediction equation in predicting future cardio-metabolic risk.

Our prediction equation was more sensitive and specific than HOMA-IR in predicting future CVD event. One obvious explanation is that the prediction equation incorporates measures of central adiposity and triglycerides, which are both associated with insulin resistance and are independent risk factors for CVD. To better understand this, we compared this prediction equation with metabolic syndrome (whose defining criteria include three out of the following five above predefined cut-offs – waist circumference, triglyceride, HDL cholesterol, fasting glucose and blood pressure) in predicting future CVD. We found that the prediction equation had significantly higher sensitivity over metabolic syndrome, and was independently associated with a four-fold increase in the odds of future CVD. This indicates that the improved prediction of CVD is due to obligatory inclusion of WHR and TG in our equation, which allows for bothbetter approximation of insulin resistance states, and improved prediction of CVD. Additionally, the prediction equation uses continous variables while metabolic syndrome uses binary variables, which may also explain greater predictive accuracy for our equation compared to metabolic syndrome. This equation could provide a closer estimate for insulin sensitivity in predicting CVD and DM risk especially in population studies. Unlike the metabolic syndrome classification or available scoring systems, the prediction equation also provides an independent continuous variable which can be utilised for epidemiological research modeling.

The clinical utilty and strength of our study is that we have developed an estimation for insulin sensitivity that was able to predict both both future DM and CVD in an independent cohort. One limitation is that CVD was defined by self-report and that definitions of CVD were not identical at baseline and follow up. However, we have no reason to suppose that this would affect one test more than the other. Moreover, the area under the ROC curve for HOMA-IR for prediction of DM and CVD in our study is similar to that reported elsewhere [25].

In summary, we show that fasting triglycerides and waist hip ratio are important determinants of insulin sensitivity, and can be combined with fasting insulin levels to provide a more accurate estimation of insulin sensitivity, and better prediction of future risk of CVD than HOMA-IR.

## Supporting Information

Table S1 **Generation of prediction equation in SAMS.**
(DOCX)
