# Supplementary material for: New Measure of Insulin Sensitivity Predicts Cardiovascular Disease Better than HOMA Estimated Insulin Resistance
Source: PLoS One. 2013 Sep 30;8(9):e74410. doi: 10.1371/journal.pone.0074410 (PMC3787028; doi:10.1371/journal.pone.0074410)
Supplement: Table S1 — Generation of prediction equation in SAMS. (DOCX) [file pone.0074410.s001.docx]

Table S1. Generation of prediction equation in SAMS

| **Parameter** | **B** | **Bias** | **SE** | **95% CI^a^** | | ***P*** |
| --- | --- | --- | --- | --- | --- | --- |
| **Insulin^b^** | -0.39 | 0.02 | 0.1 | -0.62 | -0.23 | <0.001 |
| **Triglyceride ^b^** | -0.21 | 0.01 | 0.13 | -0.53 | -0.01 | 0.034 |
| **Waist hip ratio^b^** | -2.63 | -0.04 | 1.14 | -4.6 | -0.26 | 0.004 |
| **Constant** | 2.65 | -0.05 | 0.35 | 2.1 | 3.52 | <0.001 |

Dependent variable - insulin sensitivity by clamp^b^; adjusted R^2^ = 0.58

^a^ = Bias-corrected and accelerated confidence intervals

^b^ = log-transformed variable

SAMS – Singapore Adult Metabolism study
